# A mesoscopic stochastic model for the specific consumption rate in substrate-limited microbial growth

**DOI:** 10.1371/journal.pone.0171717

**Published:** 2017-02-10

**Authors:** F. J. Arranz, J. M. Peinado

**Affiliations:** 1 Grupo de Sistemas Complejos, Universidad Politécnica de Madrid (UPM), av. Puerta de Hierro, 2 - 4, 28040 Madrid, Spain; 2 Departamento de Microbiología III, Facultad de Biología, Universidad Complutense de Madrid (UCM), c. José Antonio Nováis, 2, 28040 Madrid, Spain; 3 CEI Campus Moncloa, UCM-UPM, Madrid, Spain; University of Szeged, HUNGARY

## Abstract

The specific consumption rate of substrate, as well as the associated specific growth rate, is an essential parameter in the mathematical description of substrate-limited microbial growth. In this paper we develop a completely new kinetic model of substrate transport, based on recent knowledge on the structural biology of transport proteins, which correctly describes very accurate experimental results at near-zero substrate concentration values found in the literature, where the widespread Michaelis-Menten model fails. Additionally, our model converges asymptotically to Michaelis-Menten predictions as substrate concentration increases. Instead of the single active site enzymatic reaction of Michaelis-Menten type, the proposed model assumes a multi-site kinetics, simplified as an apparent all-or-none mechanism for the transport, which is controlled by means of the local substrate concentration in the close vicinity of the transport protein. Besides, the model also assumes that this local concentration is not equal to the mean substrate concentration experimentally determined in the culture medium. Instead, we propose that it fluctuates with a mostly exponential distribution of Weibull type.

## Introduction

Due to the microscopic nature of single microbial cells, the knowledge and mathematical modelling of the mechanisms controlling growth of microbial populations is considered essential to analyze, predict, and control the effect of microbes in nature and industry. Among the factors controlling microbial growth, its dependence on the concentration in the medium of the carbon and energy source is recognized as one of the most relevant ones.

The equation developed by Monod [1], which describes a hyperbolic dependence of the specific growth rate on the external concentration of the carbon source, continues to be the most widely used model in biotechnology and applied microbiology. However, Monod equation is a purely empirical model where the model parameters do not have a clear physiological meaning. For this reason, many attempts have been developed to give a mechanistic structure to the hyperbolic Monod equation. Liu [2] revised these attempts, concluding that “no universal physical meaning of the Monod constant can be revealed”.

Since cell growth is a very complex process involving thousands of metabolic reactions, modelling its dependence on the carbon source using a single equation implies gross oversimplifications. One of the simplifications that have been more successful is based on the concept of transport-limited growth [3] that attributes the control of the growth rate exclusively to the transport rate of substrate into the cell. This approach is strongly based on the fact that both the specific growth rate and the substrate consumption rate seem to depend on the external substrate concentration in a way which can be described by a hyperbolic equation, identical to the Michaelis-Menten model for enzyme-catalyzed reactions [4]. Even in some cases it has been found experimentally that the two apparent affinity constants (defined as the substrate concentration at which both the growth rate and the consumption rate are half maximum) present close numerical values and, even more, that substrate chemical analogues inhibit both substrate consumption and growth in the same way and with a similar inhibitory constant [3].

However, there is also experimental evidence that this is not always the case (see Button [5] for a review). A discrepancy of great ecological relevance is the experimental detection of a threshold substrate concentration below which no growth occurs, whereas Michaelis-Menten kinetics predicts always a monotonous increase of the rate with the increase of substrate. Other limitations of the models based on the Michaelis-Menten kinetics are observed when the transport process is studied isolated, in resting cells or vesicles. In some cases it is observed that carrier-mediated transports that should display saturation kinetics show a linear dependence on substrate concentration, behaving as gated channels (reviewed by Conde et al. [6] and Naftalin [7]). New assumptions and mathematical modifications have been introduced to explain these discrepancies between the simple hyperbolic model of Monod and the more complex relations experimentally observed, but usually those models maintain the basic hyperbolic structure [5, 8, 9].

Based on those discrepancies, we think that simple Michaelis-Menten kinetics should not be applied, in general, to transport processes, and that the specific characteristics of the transport proteins, as compared with soluble enzymes, should be taken into account. Traditionally the transport processes have been considered an enzymatic reaction just because “they comprise a single major substrate-binding site interacting specifically with a single substrate molecule in each transport cycle” (Diallinas [10]). However, even if transport is considered an enzyme-catalyzed reaction, it is catalyzed by an immobilized enzyme and it was recognized from the beginning of immobilization studies that the substrate concentration in contact with the immobilized enzyme was always lower that its macroscopic concentration in the bulk medium [11]. Besides, genetic, kinetic and structural data have revealed the presence in the carrier proteins of secondary binding sites, interacting transitorily with substrate molecules, that would participate in the opening and closing of the carrier, facilitating the transport of the substrate molecule bind to the primary or major binding site. Although the helping substrate molecules do not cross the membrane, they do participate in the transport process and should be included in the kinetic model [6, 7, 10].

The development of models of substrate-limited growth had been hindered by the lack of accuracy in the experimental data on substrate concentration under that conditions [12]. Fortunately, the recent design of a new culture system, the retentostat, by the group of Prof. Pronk, have provided accurate experimental data for *Saccharomyces cerevisiae* cultures at near zero growth rates at very low concentrations of substrate [13, 14]. Clearly their results are incompatible with any convex curve at near-zero substrate concentration, and hence with the convex hyperbolic curve derived from Michaelis-Menten kinetics. In this work, we submit that the discrepancies observed between the simple Michaelis-Menten hyperbolic model (or any convex curve model) and the data obtained by the group of Prof. Pronk could be explained if the conditions derived from the immobilization of the carrier inside the membrane, as well as new knowledge on the structure of the transport system, were introduced in the model.

The estimation of the substrate concentration in the vicinity of the transport protein has been attempted by several mathematical models generally based on the diffusion of the substrate molecule. However due to the different types of cell structures, cell walls and cell cultivation that can be found, we think that a general diffusion-based model would be difficult to obtain. Instead, we propose that the heterogeneous distribution of molecules close to the transport protein could be described by a probability distribution of Weibull type. We propose also that the interaction of several substrate molecules with the carrier would be described more accurately by a Hill model equation.

Consequently, we have derived our model on a mesoscopic description of this local environment, calculating substrate concentration close to the carrier as a probability and using also a simplification of Hill kinetics for the transport step based on a limited number of participating substrate molecules. Thereby, the resulting model has the correct concave behaviour at near-zero substrate concentration, as is suggested by the experimental data obtained by the group of Prof. Pronk [13, 14], fitting accurately these data, especially those obtained in the retentostat with high accuracy [14].

## Results

### Substrate-limited microbial growth

Three parameters are standard in substrate-limited cell growth analysis, the specific growth rate *μ*, the specific consumption rate *q*, and the yield *Y*, defined by means of

$$\begin{array}{c} \mu \equiv \frac{1}{N}\frac{dN}{dt}, \end{array}$$ (1)

$$\begin{array}{c} q\equiv -\frac{1}{N}\frac{dC}{dt}, \end{array}$$ (2)

$$\begin{array}{c} Y\equiv -\frac{dN}{dC}, \end{array}$$ (3)

where *N* is the concentration of cells, *C* is the concentration of nutrient substrate, and *t* is the time. Observe that above equations lead to

$$\begin{array}{c} \mu =q\cdot Y, \end{array}$$ (4)

which is a fundamental equation describing the substrate-limited growth as a process whose velocity *μ* depends on two factors: The rate *q* at which substrate is consumed, and the efficiency *Y* at which the consumed substrate is transformed by the cell metabolism into new biomass.

Considering fixed amount of substrate, fixed external conditions (pressure, volume, temperature, and pH), and also no volume limitations (non-saturated liquid medium), it is assumed that the specific growth rate is a function *μ*(*C*) of the substrate concentration *C*, and that the specific consumption rate is likewise a function *q*(*C*) of the substrate concentration. Moreover, it is generally accepted that not all consumed substrate is devoted to the synthesis of new biomass, but part of it is used to maintain alive the existing cells. Accordingly, the ratio between the substrate consumption devoted to maintenance and that devoted to growth will affect the yield value. However, due to the usually small value of the specific maintenance consumption rate, this effect can be neglected and then the yield can be assumed mainly constant.

The most extended model for the specific growth rate *μ*(*C*) is the purely empirical model from Monod [1], which has the hyperbolic form

$$\begin{array}{c} \mu \left(C\right)=\mu_{\text{max}}\frac{C}{K+C}, \end{array}$$ (5)

with constant *μ*_max_ and *K*. This model acquires physiological meaning by assuming that the nutrient transport through cell membrane follows the Michaelis-Menten enzymatic kinetics [4], namely *v* = *v*_max_*C*/(*K* + *C*), so that, since *v* = −*dC*/*dt*, the specific consumption rate, according to the definition in Eq (2), will be given by

$$\begin{array}{c} q\left(C\right)=q_{\text{max}}\frac{C}{K+C}, \end{array}$$ (6)

being *q*_max_ = *v*_max_/*N* the maximum specific consumption rate reachable by each cell, and *K* the Michaelis-Menten constant. Thus, from the Michaelis-Menten based model in Eq (6), the relationship in Eq (4) leads the Monod model, with *μ*_max_ = *q*_max_ ⋅ *Y*.

Some generalizations of the original Monod model (and then of the associated Michaelis-Menten based model), many of them without underlying physiological meaning, have been proposed (see, e.g., Roels’ book [12] for a review), as well as other models based on non-equilibrium thermodynamics [15] giving a logarithmic form for the specific growth rate. All these models have a common feature, namely, the derivative at null concentration *C* = 0 is not null, that is, (*dq*/*dC*)_0_ ≠ 0, taking generally its maximum value rather than zero value at *C* = 0. At this point, we introduce our mesoscopic stochastic model for the specific consumption rate, which has the distinctive feature (*dq*/*dC*)_0_ = 0 and, what is more interesting, implications on the mechanism of transport of nutrients through cell membrane.

### Specific consumption rate model

In order to establish the model for the specific consumption rate, the following two assumptions are considered:

1. The local substrate concentration, in the immediate neighbourhood of the corresponding membrane transport protein, fluctuates around the mean concentration (bulk concentration) with high probability for concentration below the mean and with low probability for concentration above the mean.
2. The substrate penetrates cell membrane if and only if the local substrate concentration, in the immediate neighbourhood of the transport protein, reaches or exceeds certain concentration threshold which will be named as *activation concentration*. Then, the substrate penetrates cell membrane at constant rate.

**Fig 1:**
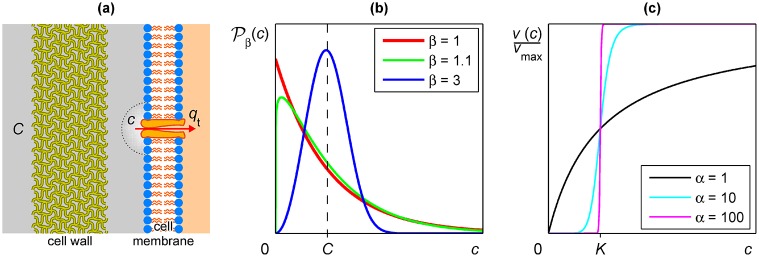
Assumptions in the proposed model. **(a)** Schematic representation of the cell interface in the neighbourhood of a transport protein, with consumption rate *q*_t_, showing the concept of local substrate concentration *c* versus bulk concentration *C*. **(b)** Probability density function $P_{\beta }\left(c\right)$ corresponding to the Weibull distribution with mean value *C* and different values of parameter *β*. Note that *β* = 1 corresponds to the exponential distribution. **(c)** Normalized reaction rate *v*(*c*)/*v*_max_ corresponding to the Hill equation with constant *K* and increasing values of the interaction coefficient *α*. Note that *α* = 1 case corresponds to the Michaelis-Menten equation.

The first assumption concerns the features of substrate solution in the neighbourhood of the corresponding transport protein. As is represented in Fig 1a, substrate at bulk concentration *C* is transported into the cell by the corresponding protein with rate *q*_t_, so that local substrate concentration *c* in the immediate neighbourhood of the transport protein will decrease. Forced convection in the liquid medium would immediately restore bulk concentration, but the existence of the cell wall prevents forced convection, so that bulk concentration will be restored by means of diffusion. Since substrate diffusion is a very slow process, it seems reasonable that the local concentration is smaller than bulk concentration with high probability, and greater than bulk concentration with low probability. The exponential distribution is the simplest probability distribution with these features among other suitable features. However, exponential distribution has its maximum probability density at the perhaps unrealistic value *c* = 0, so that we can improve the model by using the more general Weibull distribution, which includes the exponential distribution as a particular case.

The probability density function $P_{\beta }\left(c\right)$ corresponding to the Weibull distribution is given by

$$\begin{array}{c} P_{\beta }\left(c\right)={\left(\frac{A_{\beta }}{C}\right)}^{\beta }\beta c^{\beta -1}\exp[-{\left(A_{\beta }\frac{c}{C}\right)}^{\beta }], \end{array}$$ (7)

where *A*_*β*_ = Γ(1 + 1/*β*), being Γ(*x*) the Gamma function or generalized factorial of (*x* − 1), *c* ∈ [0, ∞) is the local substrate concentration in the neighbourhood of the transport protein, $C=\int_{0}^{\infty }c\;P_{\beta }\left(c\right)\;dc$ is the mean value of the distribution or bulk concentration, and *β* ∈ [1, ∞) is the shape parameter. Notice that for *β* = 1 the factor *A*_*β*_ in Eq (7) fulfils *A*_1_ = 1, and then the exponential distribution is obtained

$$\begin{array}{c} P_{1}\left(c\right)=\frac{1}{C}\exp(-\frac{c}{C}). \end{array}$$ (8)

The Weibull distribution is depicted in Fig 1b for increasing values of *β* parameter. Observe that for *β* near to unity, the distribution satisfies the first assumption in our model with its maximum probability density at a concentration value greater than zero. At higher values of *β*, the distribution takes a normal-like shape, breaking the first assumption, so that only near to unity values will be suitable. In the limit *β* → ∞, the variance, given by *σ*^2^ = *C*^2^(*A*_*β*/2_/*A*_*β*_ − *A*_*β*_), tends to zero, the Weibull distribution tending then to the Dirac delta distribution centred at mean concentration *C*.

The second assumption concerns the features of the mechanism of transport. New and relevant data have been published in the last few years about the molecular mechanisms underlying the transport process. Although the classical view in transport kinetics is that a single substrate molecule interacts with a single binding site of the transport protein, the latest research on this issue [10] shows the existence of several binding sites, which, when activated, would also induce conformational changes. These additional sites should be taken into account in a kinetic model, as we have mentioned in the *Introduction*. However, they are neglected completely in the Michaelis-Menten model, which considers a single binding site. To integrate these additional sites in a kinetic model a sound alternative could be provided by the Hill equation [16], which has been widely used in allosteric enzyme kinetics [17], that is, in substrate-protein reactions where the enzyme has several binding sites whose activation by substrates lead to protein conformational changes, affecting the reaction rate. Another novel result in transport kinetics has been the detection of alternative channel-like gating behaviour [6, 10] in which transport rate does not increase with the substrate concentration but works at maximum velocity when the substrate concentration is above a critical value and does not work below it. Developing this approach we have found that Hill equation can also provide an explanation to this gate behaviour. Notice that the *all-or-none* mechanism of a gate can be described by the Heaviside step function, however, as is shown in Fig 1c, the Hill equation *v* = *v*_max_*c*^*α*^/(*K*^*α*^ + *c*^*α*^), with *α* ∈ [1, ∞), tends rapidly to a smoothed Heaviside step function as the interaction coefficient *α* increases (observe that for *α* = 1 Hill equation becomes Michaelis-Menten equation). So, we have simplified our model assuming that transport proteins have several binding sites, as it has been experimentally demonstrated in some cases, and consequently, the transport kinetics can be better described by the Hill equation. We also assume that the number of sites and the interaction among them is high enough to produce an apparent all-or-none mechanism where, under these conditions, the constant *K* of Hill equation corresponds to the activation concentration *c*_ac_ in our model, the substrate concentration threshold above which transport occurs.

Thus, considering both assumptions jointly, the probability *P* of finding a local concentration *c* equal or greater than the activation concentration *c*_ac_ will be

$$\begin{array}{ccc} P\{c\geq c_{\text{ac}}\} & = & \int_{c_{\text{ac}}}^{\infty }P_{\beta }\left(c\right)\;dc\\ & = & \exp[-{\left(A_{\beta }\frac{c_{\text{ac}}}{C}\right)}^{\beta }]. \end{array}$$ (9)

So that, if the substrate penetrates cell membrane through each transport protein at the constant rate *q*_t_ when the local concentration fulfils *c* ≥ *c*_ac_, and each cell has *n* transport proteins on average, then the statistically observable value of the specific consumption rate *q*(*C*) will be given by

$$\begin{array}{ccc} q(C) & = & q_{\text{max}}\cdot P\left\{c\geq c_{\text{ac}}\right\}\\ & = & q_{\text{max}}\cdot \exp[-{\left(A_{\beta }\frac{c_{\text{ac}}}{C}\right)}^{\beta }], \end{array}$$ (10)

with *q*_max_ = *nq*_t_, resulting in the general functional form for the specific consumption rate from the proposed model. Considering the particular case of the exponential distribution instead of the general Weibull distribution, corresponding to *β* = 1 and then *A*_1_ = 1 as was indicated above, the following simpler expression is obtained

$$\begin{array}{c} q\left(C\right)=q_{\text{max}}\cdot \exp(-\frac{c_{\text{ac}}}{C}). \end{array}$$ (11)

Observe that, in this model, the smooth dependence of the (macroscopic) specific consumption rate on (bulk) concentration is due to the stochastic fluctuations of concentration in the neighbourhood of the transport protein, being the dependence of the microscopic consumption rate on local concentration a non-smooth Heaviside step function (all-or-none mechanism).

**Fig 2:**
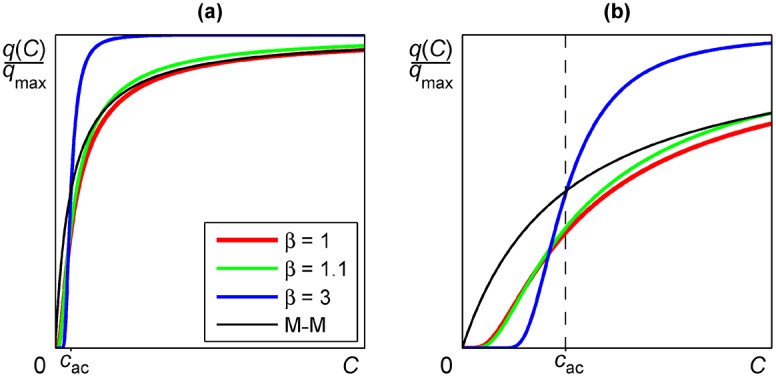
Specific consumption rate: Proposed model versus Michaelis-Menten based model. **(a)** Normalized specific consumption rate *q*(*C*)/*q*_max_ from the proposed model for an activation concentration *c*_ac_ and different values of parameter *β*, and also from Michaelis-Menten based model (M-M) assuming *K* = *c*_ac_. **(b)** Magnification of the region in panel (a) corresponding to concentration values near to zero.

The graphical representation of the specific consumption rate given in Eq (10) is depicted in Fig 2 for different values of parameter *β*, along with the curve from the Michaelis-Menten based model by assuming *K* = *c*_ac_ in Eq (6). Observe that for values of parameter *β* away from unity, corresponding to a normal-like distribution that breaks the first assumption, the curves from both models differs significantly for all concentration values. Indeed, in the limit *β* → ∞ the curve from our model tends to the Heaviside step function centred at *C* = *c*_ac_. On the other hand, for values of parameter *β* near to unity the curves from both models are virtually overlapped, differing only at concentration values near to zero, where they are clearly different. Specifically, as was indicated above, the derivative at null concentration in our model is zero [strictly, the derivative at null concentration is undefined, nevertheless the right-hand limit is zero, lim_*C* → 0^+^_(*dq*/*dC*) = 0], whilst in the Michaelis-Menten based model it takes its maximum value. Moreover, the Michaelis-Menten based model has negative second derivative (convex curve) for all concentration values, whilst our model has positive second derivative (concave curve) in the vicinity of null concentration, and negative second derivative (convex curve) for higher concentration values, the inflection point corresponding to *C* = *A*_*β*_*c*_ac_(1 + 1/*β*)^−1/*β*^. Note then that as parameter *β* increases from *β* = 1 to limit *β* → ∞, the concentration value *C* corresponding to the inflection point increases from *C* = *c*_ac_/2 to *C* = *c*_ac_.

Finally, in the same way as the Michaelis-Menten based model for specific consumption rate in Eq (6) is related to the Monod model for specific growth rate in Eq (5) by means of the fundamental relation in Eq (4), assuming constant yield, the following expression is obtained in our model for the specific growth rate

$$\begin{array}{c} \mu \left(C\right)=\mu_{\text{max}}\cdot \exp[-{\left(A_{\beta }\frac{c_{\text{ac}}}{C}\right)}^{\beta }], \end{array}$$ (12)

where *μ*_max_ = *q*_max_ ⋅ *Y*. In the particular case of the exponential distribution, that is *β* = 1 and then *A*_1_ = 1, the expression in Eq (12) is simplified to

$$\begin{array}{c} \mu \left(C\right)=\mu_{\text{max}}\cdot \exp(-\frac{c_{\text{ac}}}{C}). \end{array}$$ (13)

### Fitting the data on *S. cerevisiae* growing at very low substrate concentrations

As was stated above in the *Introduction*, the results at very low concentrations of substrate obtained by the group of Prof. Pronk [13, 14] are incompatible with the convex hyperbolic curve derived from Michaelis-Menten kinetics in Eq (6), as well as with any convex curve at near-zero substrate concentration. These results correspond to two sets of experimental data for specific consumption rate *q* versus substrate concentration *C*, both corresponding to cell cultures of the same strain of *Saccharomyces cerevisiae* carried out under identical conditions, but using different experimental techniques: The classical chemostat device, used by Diderich et al. [13], and the innovative retentostat device designed for experiments at near-zero specific growth rates, used by Boender et al. [14]. In this way, we have performed for both data sets a nonlinear fitting to the proposed model in Eq (10), including the simpler version in Eq (11), and also to the Michaelis-Menten based model in Eq (6).

**Fig 3:**
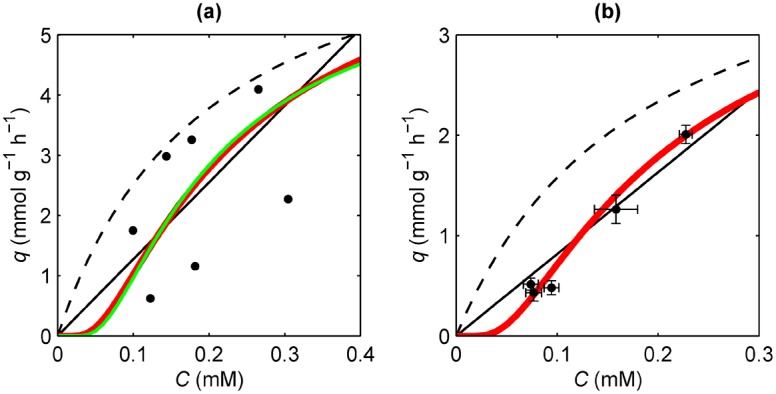
Fitting of experimental data to the Michaelis-Menten based model and to the proposed model. **(a)** Experimental values of specific consumption rate *q* versus substrate concentration *C* for *S. cerevisiae*, taken from Diderich et al. [13]. The fitted curve corresponding to the general—Weibull—proposed model (green line), as well as the curve corresponding to the simpler—exponential—version (red line), along with the fitted curve from the Michaelis-Menten based model (black line) are also depicted. Notice that Michaelis-Menten curve degenerates into a straight line. Additionally, the Michaelis-Menten curve corresponding to the fitting parameters from the simpler—exponential—version of the proposed model, assuming *K* = *c*_ac_, has also been represented (dashed black line). **(b)** Same as described for panel (a) but with the experimental data taken from Boender et al. [14]. In this case the fitting to the general—Weibull—proposed model corresponds to the simpler—exponential—version.

The chemostat data at near-zero substrate concentration from Diderich et al. [13] are shown in Fig 3a. As is well known, and also evidenced by Diderich et al. [13], the physiology of *S. cerevisiae* exhibits a dual behaviour. Namely, a purely respiratory mechanism at low growth rates (*μ* < 0.3 h^−1^) with high yield, and a mixed mechanism (respiration and alcoholic fermentation, simultaneously) at high growth rates (*μ* > 0.3 h^−1^) with low yield. In order to test our model versus the Michaelis-Menten based model at near-zero substrate concentration, only data corresponding to high yield mechanism have been used. Note that these data at very low concentration values have a certain degree of dispersion, and then no high accuracy. Observe that this experimental data set implies positive second derivative (concave curve) for the function *q*(*C*) in the vicinity of null concentration, so that the strictly convex hyperbola (negative second derivative for all concentration values) from the Michaelis-Menten based model fails in the fitting process, degenerating into a straight line with slope *b* = 12.74 mmolg^−1^ h^−1^ mM^−1^, and leading to infinite values for fitting parameters *K* and *q*_max_. However, the proposed model fits this data set reasonably well, due to the existence of its inflection point. As is shown in Table 1, where the corresponding fitting parameters and fitting quality parameters are indicated, both the general—Weibull—expression and the simpler—exponential—version of the proposed model, lead to nearby fitting parameters with similar fitting quality (see *Methods* section for detail on fitting process). Note that fitted parameter *β* from the Weibull distribution is close to unity (*β* = 1.21), so that the corresponding probability distribution will be close to the exponential distribution.

**Table 1:** Comparison of the fitting of experimental data by using the Michaelis-Menten based model and the proposed model.

	Diderich et al. data^a^			Boender et al. data^b^		
	Michaelis-Menten	Proposed model		Michaelis-Menten	Proposed model	
	based model	exponential	Weibull	based model	exponential	Weibull
*β*	—	—	1.21	—	—	1.00
*K*, *c*_ac_ (mM)	∞	0.200	0.181	∞	0.184	
*q*_max_ (mmolg^−1^ h^−1^)	∞	7.56	6.44	∞	4.48	
$\langle R_{C}^{2}\rangle$ (mM^2^)	6.93 × 10^−3^	5.77 × 10^−3^	5.62 × 10^−3^	4.65 × 10^−4^	8.95 × 10^−5^	
〈|*R*_*C*_|〉 (mM)	7.91 × 10^−2^	6.93 × 10^−2^	6.76 × 10^−2^	1.85 × 10^−2^	7.99 × 10^−3^	
|*R*_*C*_|_max_ (mM)	1.26 × 10^−1^	1.38 × 10^−1^	1.40 × 10^−1^	3.55 × 10^−2^	1.31 × 10^−2^	
$\langle R_{q}^{2}\rangle$ (mmol^2^ g^−2^ h^−2^)	1.13	1.14	1.15	3.09 × 10^−2^	1.31 × 10^−2^	
〈|*R*_*q*_|〉 (mmolg^−1^ h^−1^)	1.01	1.01	1.01	1.51 × 10^−1^	9.55 × 10^−2^	
|*R*_*q*_|_max_ (mmolg^−1^ h^−1^)	1.61	1.65	1.66	2.90 × 10^−1^	1.59 × 10^−1^	

^a^ Data from reference [13].

^b^ Data from reference [14].

Moreover, the highly accurate retentostat data at near-zero substrate concentration from Boender et al. [14] are shown in Fig 3b, along with the fitted curves from Michaelis-Menten based model and proposed model. In this case the fitted parameter *β* decreases to minimum value *β* = 1, so that the general Weibull distribution coincides to the simpler exponential distribution. Observe that this highly accurate data set strongly implies positive second derivative (concave curve) in the vicinity of null concentration, leading to the failure of the Michaelis-Menten based model, which degenerates into a straight line with slope *b* = 8.15 mmolg^−1^ h^−1^ mM^−1^. The fitting parameters and the corresponding fitting quality parameters are indicated in Table 1, where it is noteworthy the low values of the quality parameters, that is, the high quality of the fitting to the proposed model.

## Discussion

The hyperbolic dependence of the specific growth rate on the external substrate concentration, empirically found by Monod, is explained by the substrate-limited growth hypothesis applying two main assumptions. The first assumption is that the rate of growth would depend mostly on the rate of consumption of the limiting substrate, when the yield factor is constant and maintenance energy is not significant. The second is that the rate of substrate consumption would depend on the rate of membrane transport of that substrate, and transport thorough a carrier would display simple Michaelis-Menten kinetics. So, growth rate dependence on the substrate is hyperbolic because the transport rate dependence on substrate concentration is hyperbolic.

Some relevant examples of discrepancies found between the predicted hyperbolic behaviour and experimental results, found in natural and laboratorial environments, have been given in the *Introduction*. Attempts to found better mathematical models have been based on additions to the basic Michaelian equation, generally designed to improve the fitting capacity of the model [2]. Our approach in this work has been completely different. We have tried to introduce into the structure of the model some knowledge on the transport process that, either had been neglected in those previous attempts or has been recently acquired.

In our approach, we gave great relevance to the microenvironment surrounding the carriers that are proteins immobilized in some definite places into the cytoplasmic membrane. The immobilization, and its consequences, has not been introduced in most of the previous models, which generally consider carriers as enzymes in suspension, surrounded by a concentration of substrate displaying a normal-like distribution around the mean. That mean would be numerically equal to the macroscopic concentration experimentally determined in the external medium. In contrast with those *homogeneous* models, we claim that the distribution of substrate molecules around the carriers does not follow a normal-like distribution, and that the external structures of the cells and their physiology have a great importance in the distribution. We propose that this distribution can be described by the Weibull distribution in Eq (7), where the parameter *β* would be a measure of the influence of the transport system structure on the relation between the actual concentration of substrate close to the carrier *c* and the macroscopic concentration in the medium *C*. In Fig 1b we can observe that when *β* is equal or close to unity, the structure of the transport environment has a great influence, and the most probable concentration to be found close to the carrier is zero or near zero, far from the macroscopic concentration. In contrast, with a *β* value of three, the most probable concentration is close to the macroscopic concentration.

In our model, as in most others previously published, we postulate that the transport process can be considered an enzyme-catalyzed reaction and as such, would display Michaelian kinetics. However, as recent structural and genetic studies cited in the *Introduction* have shown, there are secondary active sites in the carriers and more than one substrate molecule participates in the process. Due to this fact the transport can not be described by simple Michaelian kinetics. Instead, the Hill equation has to be applied. In this equation, the interaction coefficient *α* depends on the number of secondary active sites and on the interactions among them. When *α* is high, and this seems to be the case by the number of active sites that have been experimentally estimated in some cases [6, 7, 10], the sigmoid Hill curve tends to the Heaviside step function, as is shown in Fig 2b. This means that below a narrow range around a concentration of substrate equal to the affinity constant there is no transport and above this narrow range transport occurs at its maximum rate. In this last case the carrier will function as if it were a gated channel, a behaviour that has been experimentally observed in some cases [6, 10].

We think that, in most of the published experiments, the interaction coefficient must be high and those carriers with substrate concentrations above the affinity constant will be the only open, transporting substrate at their maximum velocity. In that conditions the substrate concentration in the microenvironment of the carriers became the only variable determining changes in transport and that explains how those Michaelian transport systems do not displays their own hyperbolic kinetic but the sigmoid-like dependence described by the probability distribution of the substrate molecules around the carriers.

The proposed model is partly based on the mostly exponential fluctuations of the substrate concentration in the immediate neighbourhood of the transport protein. However, the maximum probability density for the exponential distribution is at null concentration, as is shown in Fig 1b. So that, to obtain a perhaps more realistic description, a Weibull distribution with shape parameter *β* ≥ 1 but close to unity was considered, whereby the maximum probability density is close to null concentration albeit it is not null. Notice that the Weibull distribution allows values for the shape parameter in the range *β* ∈ (0, ∞), the probability density at null concentration being infinite for *β* ∈ (0, 1), so that this range has been excluded in our model.

Despite using the Weibull distribution in our general model, we should highlight the simpler version obtained by assuming the exponential distribution. In this case, our model has exactly the same parameters as the Michaelis-Menten based model, assuming *K* = *c*_ac_, and the curves *q*(*C*) from both models are virtually indistinguishable except in the vicinity of *c*_ac_, i.e. at near-zero concentration, where they clearly differ, as is shown in Fig 2. Therefore, when dealing with experimental data away from null concentration, as is the case in most works, both models will give similar values for fitting parameters *c*_ac_ = *K* and *q*_max_. This is the meaning of the dashed line in panels (a) and (b) of Fig 3: It represents the hyperbolic curve obtained if higher concentration data, instead near-zero concentration data, had been considered. Clearly, for both the chemostat data from Diderich et al. and the retentostat data from Boender et al., the dashed line corresponding to this hypothetical hyperbolic curve is far away from near-zero data, suggesting the inadequacy of Michaelis-Menten based model at near-zero concentration values. Additionally, in order to highlight the simpler—exponential—version of the proposed model, it is noteworthy the excellent fitting of the general—Weibull—model with *β* = 1, that is the simpler—exponential—version, to the highly accurate retentostat data.

Moreover, one of the distinctive features of our model is the existence of an inflection point, so that the corresponding curve *q*(*C*) is concave (positive second derivative) in the vicinity of null concentration. We do not know the existence of another model with this feature in the literature. Indeed, the Michaelis-Menten based model fails in the fitting of near-zero concentration data, because the optimization process tries to obtain a curve with positive second derivative, but the hyperbolic curve has strictly negative second derivative, so that the optimization process leads to a degenerate hyperbola with null second derivative, i.e. a straight line, giving unphysical infinite values for fitting parameters *K* and *q*_max_.

We submit that our model can be very easily tested with experimental kinetic data to which simple Michaelian kinetics can not be fitted. As it is a structured model, the results could be interpreted in terms of the two assumptions underlying the model, i.e., the relevance of the cell external structures and its physiology in the determination of the distribution of substrate molecules around carriers and also the number and interaction of secondary active sites in the carriers. The results of that type of analysis should provide new hypothesis and lines of research that, hopefully, would increase our knowledge of the microbial transport process and its relation with cell growth.

## Concluding remarks

We have developed a completely new model for the specific consumption rate in substrate-limited microbial growth, which accounts for very accurate experimental results at near-zero substrate concentrations obtained with *S. cerevisiae* [13, 14]. These results suggest that the curve depicting specific consumption rate versus substrate concentration must be concave (positive second derivative) in the vicinity of zero concentration. However, both the Michaelis-Menten based model and the other proposed models in the literature lead to a convex curve (negative second derivative), so that they are unable to account for these low concentration results. Conversely, our model has the required concave behaviour at low concentrations, and additionally it asymptotically converges to Michaelis-Menten based model as concentration increases, so that our model also accounts for experimental results at standard concentration values.

The proposed model is based on two assumptions. The first assumption, which is a consequence of the immobilized condition of the transport protein, leads to a stochastic description for the substrate concentration in the vicinity of the carrier, which fluctuates around the bulk concentration according to a (mostly exponential) Weibull probability distribution. The second assumption, which is a consequence of the presence of several active sites in the carrier and the participation of several substrate molecules interacting with them [10], leads to the Hill mechanism [16, 17] with high interaction coefficient for the transport process, which is approximated by the Heaviside step function.

Finally, it is worth noting that by taking a specific model for the dependence of the yield on consumption rate (the simplest model corresponding to constant yield), an additional model for the specific growth rate is derived from our model.

## Methods

### Nonlinear fitting of experimental data

We have performed for experimental data sets of Diderich et al. [13] and Boender et al. [14] a nonlinear fitting to the proposed model, both the general expression in Eq (10) and the simpler version in Eq (11), and also to the Michaelis-Menten based model in Eq (6), by minimizing the summation *S* of the squared weighted residuals

$$\begin{array}{c} S=\sum_{i}[{\left(\frac{R_{C_{i}}}{\sigma_{C_{i}}}\right)}^{2}+{\left(\frac{R_{q_{i}}}{\sigma_{q_{i}}}\right)}^{2}], \end{array}$$ (14)

where *R*_*X*_*i*__ is the residual (difference between experimental and fitted value), and *σ*_*X*_*i*__ the standard deviation, both corresponding to the *i*-th data point of each variable *X* = *C*, *q*. Notice that error bars are not provided by Diderich et al. [13], so that the suitable values for standard deviation *σ*_*C*_*i*__ = 0.1 mM and *σ*_*q*_*i*__ = 1 mmolg^−1^ h^−1^ were considered in the fitting procedure. In order to quantify the fitting goodness, the following fitting quality parameters were calculated: Mean squared residual $\langle R_{X}^{2}\rangle$, mean absolute residual 〈|*R*_*X*_|〉, and maximum absolute residual |*R*_*X*_|_max_, all of them evaluated at minimum.

In the case of the fitting to the simpler—exponential—version of the proposed model, since the number of fitting parameters is two (*c*_ac_ and *q*_max_), the minimization was performed by calculation of the squared residual summation on a suitable mesh in the test parameter space $\left({\tilde{c}}_{\text{ac}},{\tilde{q}}_{\text{max}}\right)$, so that the behaviour of the optimization surface $S({\tilde{c}}_{\text{ac}},{\tilde{q}}_{\text{max}})$ was unveiled. By reducing mesh spacing, the minimum of the surface, corresponding to the fitted parameters, was obtained with required accuracy.

For the general—Weibull—proposed model, which has three fitting parameters (*β*, *c*_ac_, and *q*_max_), the fitting procedure was started at the result previously obtained for the simpler—exponential—version, corresponding to $S(\tilde{\beta },{\tilde{c}}_{\text{ac}},{\tilde{q}}_{\text{max}})$ with $\tilde{\beta }=1$, and then increased the value of $\tilde{\beta }$ and obtained the minimum of each surface $S(\tilde{\beta },{\tilde{c}}_{\text{ac}},{\tilde{q}}_{\text{max}})$ with fixed $\tilde{\beta }$ until the achievement of the fitted parameters.

Finally, for the fitting to the Michaelis-Menten based model, since the number of fitting parameters is two (*K* and *q*_max_), the minimization was performed in the same way as in the case of the simpler—exponential—version of the proposed model. However, in this case the optimization surface $S(\tilde{K},{\tilde{q}}_{\text{max}})$ has not a finite minimum for any of the data sets. Instead, optimal parameters (*K*, *q*_max_) asymptotically tend to infinity along the straight line

$$\begin{array}{c} {\tilde{q}}_{\text{max}}\left(\tilde{K}\right)=a+b\;\tilde{K} \end{array}$$ (15)

so that the hyperbolic curve becomes the straight line

$$\begin{array}{ccc} q(C) & = & \lim_{\tilde{K}\to \infty }{\tilde{q}}_{\text{max}}\left(\tilde{K}\right)\frac{C}{\tilde{K}+C}\\ & = & \lim_{\tilde{K}\to \infty }\left(a+b\tilde{K}\right)\frac{C}{\tilde{K}+C}\\ & = & b\;C. \end{array}$$ (16)

Notice that the intercept value *a* in Eq (15) corresponds to the minimum meaningful value that fitting parameter *q*_max_ can take for each data set (namely, *a* = 4.09 mmolg^−1^ h^−1^ for Diderich et al. data, and *a* = 2.01 mmolg^−1^ h^−1^ for Boender et al. data).
